# Access to Radiation Therapy: From Local to Global and Equality to Equity

**DOI:** 10.1200/GO.21.00358

**Published:** 2022-08-12

**Authors:** Sarbani Ghosh Laskar, Shwetabh Sinha, Rahul Krishnatry, Cai Grau, Minesh Mehta, Jai Prakash Agarwal

**Affiliations:** ^1^ Department of Radiation Oncology, Homi Bhabha National Institute, Tata Memorial Centre, Mumbai, India; ^2^ Department of Radiation Oncology and Danish Center for Particle Therapy, Aarhus University Hospital, Aarhus, Denmark; ^3^ Department of Radiation Oncology, Miami Cancer Institute, Miami, FL

## Abstract

The discipline of radiation oncology is the most resource-intensive component of comprehensive cancer care because of significant initial investments required for machines, the requirement of dedicated construction, a multifaceted workforce, and recurring maintenance costs. This review focuses on the challenges associated with accessible and affordable radiation therapy (RT) across the globe and the possible solutions to improve the current scenario. Most common cancers globally, including breast, prostate, head and neck, and cervical cancers, have a RT utilization rate of > 50%. The estimated annual incidence of cancer is 19,292,789 for 2020, with > 70% occurring in low-income countries and low-middle–income countries. There are approximately 14,000 teletherapy machines globally. However, the distribution of these machines is distinctly nonuniform, with low-income countries and low-middle–income countries having access to < 10% of the global teletherapy machines. The Directory of Radiotherapy Centres enlists 3,318 brachytherapy facilities. Most countries with a high incidence of cervical cancer have a deficit in brachytherapy facilities, although formal estimates for the same are not available. The deficit in simulators, radiation oncologists, and medical physicists is even more challenging to quantify; however, the inequitable distribution is indisputable. Measures to ensure equitable access to RT include identifying problems specific to region/country, adopting indigenous technology, encouraging public-private partnership, relaxing custom duties on RT equipment, global/cross-country collaboration, and quality human resources training. Innovative research focusing on the most prevalent cancers aiming to make RT utilization more cost-effective while maintaining efficacy will further bridge the gap.

Cancer is unequivocally regarded as one of the foremost causes of morbidity and death worldwide today.^1^ Approximately 19 million new cases of cancers are diagnosed every year globally, with an estimated 10 million deaths.^2^ Besides the burden on human resources, the financial implications of cancer are perhaps more strenuous than any other communicable/noncommunicable disease. This burden is borne at all levels of care: individual, hospital, and nation.

**CONTEXT**

**Key Objective**
To describe the global scenario of radiation therapy (RT) in terms of machinery and power with a particular focus on low-income countries and low-middle–income countries.
**Knowledge Generated**
There is increasing disparity in the distribution of resources between high-income countries/high-middle–income countries and low-income countries/low-middle–income countries, which is likely to worsen in the next decade.
**Relevance**
Innovative and pragmatic ideas, including remote training of RT personnel, encouraging public/private partnership, relaxation of custom duties, and cross-country collaborations, are likely to improve the global scenario in the near future. Careful policy framing and subsequent application tailored to the prevailing local scenario can achieve long-term goals of affordable RT for all.

Cancer care still primarily relies on three main pillars for management: surgery, systemic therapy, and radiation therapy (RT). These three modalities have witnessed significant improvements in the past century to improve patient outcomes. However, the initial cost of investment and infrastructure needed and the utility/applicability distinguish these modalities at their core. Although basic surgical and medical oncology setups require smaller initial capital and operational outlay, even a relatively simple radiotherapy delivery device such as a new telecobalt setup could cost approximately 4 million US dollars. Moreover, cancer surgery and routine chemotherapy services can be provided in existing clinical facilities without dedicated cancer care facilities with minimal additional investment. Also, existing centers with surgery and daycare facilities can be used to manage noncancer ailments.

By contrast, almost all radiotherapy equipment requires specialized construction and shielding for radiation safety purposes, a capital-intensive undertaking. Even after establishing a cancer care facility, the workforce requirement in the RT department, namely, radiation oncologist, RT technologist, medical physicist, and dosimetrist, is much more than the corresponding surgical and medical oncology departments. It is critical to recognize that the ongoing expenditure on consumables such as drug therapy is very low for radiotherapy, unlike medical oncology, but the upfront capital outlay is far more intensive.

Hence, adequate global coverage of RT can be considered the Achilles' heel of the goal for cancer care for all despite being an absolute necessity. Although ensuring uniform and unvarying cancer coverage in high-income countries (HIC) and high-middle–income countries (HMICs) such as Australia and Canada has also remained elusive to date, it is the situation in the low-income countries (LIC) and low-middle–income countries (LMIC) that is perturbing.

## WHERE DO WE STAND?

The past century has seen enormous developments in RT from the era of superficial X-rays to orthovoltage, megavoltage (MV), and more recently, particle therapy; however, only telecobalt and linear accelerators (LA) are commonly used clinically for photon treatments currently. It is estimated that around 14,000 teletherapy machines are in use globally, approximately a fivefold increase compared with 1980.^3^ In 1976, it was estimated that there were 2,365 (87.6%) telecobalt machines and 336 (12.4%) LA. The ratio has now reversed; in 2020, there are only 2,004 (13.9%) telecobalt units and 12,411 (86.1%) LA. The decline in telecobalt is attributable primarily to the cumbersome, recurring source handling requirements and potential safety concerns and the remarkable versatility of the LA. However, the rise in the number of teletherapy machines has not been uniform across the world. As per the 2013 Lancet Oncology Commission estimate, the numbers of teletherapy machines in HICs, HMICs, LMICs, and LICs are 8,911, 3,115, 1,014, and 62, respectively (2013 estimate).^4^ Hence, the number of teletherapy machines in LMICs and LICs is < 10% of the global total. There is continent-wise disparity too, with North America having nearly 2,600 RT centers (out of which > 90% is accounted for by the United States), South America having 650 centers, Europe having about 1,600 centers, Africa having 250 centers, and Asia having nearly 3,000 RT centers.^3^

There is no formal estimate available globally for the number of radiation oncologists, medical physicists, and technologists. A systematic review of the global cancer workforce reveals a median of 0.28 radiation oncologists per 100,000 population and 0.15 per 100 patients with cancer worldwide. The estimated number of radiation oncologists per 100 patients with cancer for HICs, HMICs, and LMICs were 0.25, 0.19, and 0.04, respectively.^5^ No data for LICs were reported. Formal country-wise estimates for radiation oncology workforce are available for multiple countries. For example, there are an estimated 5,000 radiation oncologists in the United States for approximately 1.8 million new cancer cases diagnosed every year. The HERO studies in Europe have estimated about 10,000 radiation oncologists for an estimated 3.7 million new patients annually.^6^ In India, there are 5,000 radiation oncologists for an approximate annual incidence of 1.4 million new cases. The estimated number of radiation oncologists is < 200 for an annual estimated cancer incidence of about one million.^7^

The Directory of Radiotherapy Centres (DIRAC) registry enlists 3,318 brachytherapy cancer centers in the world. By country, the United States again has the highest number of brachytherapy centers with 772 machines. Europe has an estimated 1,100 machines, with four countries (Germany, France, Spain, and Russia) accounting for more than 50% of the brachytherapy capacity. India has 317 brachytherapy centers, while Africa has 105 brachytherapy centers, with Egypt and South Africa accounting for 47 of them. As per a recent report, as of March 2020, only 21 (39%) out of 54 countries in Africa have access to brachytherapy.^8^

A global estimate of the number of computed tomography (CT) simulators and treatment planning systems is challenging to garner as no formal data exist for most of the continents. Documenting the distribution of radiotherapy departments and the availability of radiotherapy equipment in the European countries is an integral part of HERO—the ESTRO Health Economics in Radiation Oncology project. As per the HERO report published in 2014, 2,192 LA, 96 dedicated stereotactic machines, and 77 cobalt machines were available in the 27 countries.^9^ A total of 12 countries had at least one cobalt machine in use. There was a median of 0.5 simulator per MV unit (range, 0.3-1.5) and 1.4 (range, 0.4-4.4) simulators per department. Of the 874 simulators, 654 (75%) were capable of 3D imaging (CT scanner or cone beam computer tomography option). The number of MV machines (cobalt, LA, and dedicated stereotactic machines) per million inhabitants ranged from 1.4 to 9.5 (median, 5.3) and the average number of MV machines per department ranged from 0.9 to 8.2 (median, 2.6). The average number of treatment courses per year per MV machine varied from 262 to 1,061 (median 419). Although 69% of MV units were capable of intensity modulated radiotherapy (IMRT), only 49% were equipped for image guided radiotherapy (IGRT). There was a clear relation between socioeconomic status, as measured by gross national income per capita, and the availability of radiotherapy equipment in the countries. In many LICs in Southern and Central-Eastern Europe, there was minimal access to radiotherapy and especially to equipment for IMRT or IGRT. The European average number of MV machines per million inhabitants and per department was better, in line with QUARTS recommendations from 2005, but the survey also showed significant heterogeneity in the access to modern radiotherapy equipment in Europe, and several countries were facing substantial shortages of both equipment in general and especially machines capable of delivering high-precision conformal treatments (IMRT, IGRT). Compared with this report, the entire country of India has 90 conventional and CT-based simulators; the entire African continent, on the other hand, has < 100 simulators.^10^

The DIRAC registry lists 110 proton therapy centers globally, with the United States (37) and Japan (23) having the highest number of facilities.

## IS THIS ADEQUATE?

GLOBOCAN 2020 estimates the annual global new cancer incidence to be 19,292,789. This is expected to rise to approximately 23 million cases by 2030. Some common cancers' estimated RT utilization rate is as follows: breast: 87%, cervical: 71%, head and neck: 84%, lung: 77%, and prostate: 58%. By 2030, although the global estimate of cancer incidence is expected to rise by 50% compared with 2012, the proportionate increase in cases in the LMICs and LICs would be nearly 80%-90%, much higher than the worldwide estimates.^4^ The most common cancers expected to rise worldwide are prostate and lung cancer in men, and breast and endometrial cancers in females. All these cancers have a high RT utilization (> 50%) and are likely to accentuate the existing shortfall significantly. The estimated global shortfall of teletherapy machines is expected to rise by more than 10,000 units by 2030.

Various criteria for assessing the adequacy of teletherapy machine distribution exist. The WHO recommends one teletherapy machine for a population of one million.^11^ This has been considered inadequate by many subsequent reports.^9,12^ Even with this conservative estimate, 95 of 167 countries have an insufficient number of external-beam radiation therapy (EBRT) machines. Overall, 36 countries do not have any RT access within the country.^13^

Yap et al used the Collaboration for Cancer Outcomes Research and Evaluation radiotherapy utilization rate model to estimate demand for teletherapy machine in each country by estimating the number of patients in each country requiring RT by cancer type. The DIRAC was used for the actual number of teletherapy machine in each country. Using this demand-supply metric, in LICs, LMICs, UMICs, and HICs, a total deficit of 781, 2,066, 2,657, and 1,537 machines was estimated. Compared with 2002, the deficit in LICs and LMICs had increased (739 and 2,064, respectively), whereas the deficit in HMICs and HICs had decreased. Globally, < five countries had adequate teletherapy machines for the number of new cancer patients diagnosed, and the majority of Asia, eastern Europe, and Africa had large deficits.

The estimated shortfall in India for teletherapy machines is around 1,200 as per the WHO estimate.^6^ If one were to attempt to realize the ambitious goal of a global average of about four teletherapy machines for one million population, the estimated deficit in India is nearly 5,000 machines.

The global shortfall for brachytherapy is more challenging to estimate. Although the incidence of uterine cervical cancer has declined in most parts of the world (age standardised ratio [ASR]: 13.3), the incidence continues to rise in high-risk populations in Africa (ASR > 40 in some regions). It has been argued that cervical cancer is perhaps the only common cancer requiring brachytherapy as an essential curative treatment. Chopra et al^14^ suggested that one brachytherapy machine is needed for every 100 new cervical cancer patients diagnosed. By this estimate, India, with an ASR of approximately 14 for cervical cancer, had a shortfall of 127 brachytherapy machines. Although formal population incidence and utilization estimates are unavailable for the majority of the countries of the world, the severe shortfall of brachytherapy machines in LICs and LMICs in Asia/Africa and South America with the highest incidence of cervical cancer is a cause for concern. In a review of global cervical cancer incidence and mortality rates by Singh et al,^15^ a 0.2-unit increase in Human Development Index was associated with a 20% decrease in cervical cancer risk and a 33% decrease in cervical cancer mortality risk. Although this can be attributed to diagnosis and socioeconomic status factors, a shortage of brachytherapy facilities in countries with low Human Development Index score may also have significantly contributed to this.

The issue of the radiation oncology workforce is quite complicated. Concerns about an oversupply of radiation oncologists and saturation of the job market are growing in countries such as the United States and Canada. The United States reported that 14% of radiation oncology residency positions were unmatched in the 2020 match (compared with 0% in previous years) primarily because of concerns regarding job opportunities.^16^ Similar concerns are being raised in India too, with around 600 radiation oncologists graduating annually.^17^ However, the majority of the world, particularly LICs/LMICs, suffers from a lack of trained radiation oncology workforce. More than 40 countries in Asia and Africa do not have even one trained radiation oncologist. The dearth of trained medical physicists, RT technologists, and dosimetrists is likely to be far worse. Even in countries with an adequate workforce, the quality of training and service provided is worrisome and may vary.

A less well quantifiable parameter is the uniformity of distribution within the country/region. Although absolute numbers of teletherapy machines/setups do shed some light on the clustering of services, the population and geographical area should also be considered. For example, approximately 40% of the LA in Canada are located in Ontario.^18^ However, Ontario also accounts for nearly 40% of Canada's population. By contrast, about 60% of teletherapy machines in India are located in southern and western India.^19^ In an estimate for the shortfall of RT resources for cervical cancer, the majority of northern and eastern states had a severe deficit as per the overall incidence of cervical cancer in that state.^14^ Another study from 2018 reported that eight metro cities in India, constituting only 10.9% of the national population, benefit from 38% of the total RT capacity of India.^20^ Clustering is even more glaring in Africa, with a significant part of sub-Saharan Africa deprived of even basic radiotherapy facilities.^10^ In Europe, the Radiation Therapy for Cancer: Quantification of Radiation Therapy Infrastructure and Staffing Needs (QUARTS) project and other recent analyses have emphasized the disparity across the continent ranging from 2 MV EBRT units per million in Albania and Bulgaria, to more than 8 MV units per million in Belgium, Denmark, and Norway.^9^

## WHAT NEEDS TO BE DONE?

Cancer is likely to emerge as the commonest preventable cause of death in the next two decades. With nearly 60% of patients requiring RT with curative intent, the importance of expanding RT infrastructure and coverage to ensure appropriate distribution of these facilities cannot be understated.^21^ A simulation-based analysis of 5-year survival gains of 11 common cancers estimated that expanding access to radiotherapy will lead to survival gains of 2.5%-3.4% in LICs and 2.4%-6.1% in LMICs.^22^ Similarly, a simulation model estimated that expanding access to radiotherapy can lead to a 1.5% gain in 5-year breast cancer survival rates globally, with LICs (2.4%) and LMICs (5.8%) standing to benefit the most.^23^ Although global initiatives by organizations such as IAEA are essential to initiate the process, a strategy tailored for the needs of a particular geographic region is necessary for sustaining the effort. This warrants joint efforts from specialists, policymakers, governments, aid organizations, patient representatives, equipment manufacturers, and organizations such as the IAEA, ASTRO, ESTRO, and FARO. Commendable efforts have already been undertaken by the Union for International Cancer Control (UICC), which formed the Global Task Force for Radiotherapy on Cancer Control (GTFRCC) for assessing the cost of uniform RT coverage.^24^ Consequently, the Global Impact of Radiotherapy in Oncology (GIRO) project is underway and aims to use data from projects such as HERO and GTFRCC into pragmatic actions for equitable RT distribution globally.^25^

We list a few measures that countries can adopt with a focus on an LMIC such as India, where although the number of RT facilities is increasing, it is unable to keep pace with the rise in cancer incidence, and there exists disparity in the distribution and access to these services.

### Formation of Government Bodies to Identify the Problem

The one-size-fits-all approach cannot curb the spiraling global radiotherapy crisis. There is a significant disparity between countries clubbed within the LIC/LMIC group and within the country itself. Many LICs/LMICs already have more than 100 teletherapy machines (eg, India and Pakistan) and a rapidly increasing number of radiation oncologists. By contrast, > 20 countries in Africa do not have a single RT center, and most countries do not have a single native radiation oncologist. Within the HICs/HMICs, the United States has 2,555 RT centers, whereas there are only 52 in Canada, despite its vast size. Hence, a committee constituting government policymakers, administrators, investors, industry representatives, and radiation oncologists should be formed at the national level to identify the magnitude and scope of the problem, propose solutions, and devise pragmatic implementation strategies.

### Adoption of Indigenous Technology

The biggest hurdle for the shortfall in RT is fiscal. The upfront investment is the primary deterrent for establishing new RT centers. One proposed way out is to develop and propagate indigenous technology that could be more economical and can circumvent issues of applicable taxation (eg, custom duties). An excellent example is the Bhabhatron and Bhabhatron II telecobalt machines developed by the Bhabha Atomic Research Centre and Panacea Medical in India. More than 100 cost-effective units have been delivered to multiple countries, including Nepal, Madagascar, Mongolia, Tanzania, and Kenya.^26^ The same can also be extended to developing indigenous simulators and LA. Of course, one could also suggest that local taxation on life-saving medical devices is retrogressive and archaic and should be immediately abolished, as discussed below. Furthermore, as is the case with the global expansion of automobile manufacturing, incentives for global companies for local manufacturing could also go a long way.

### Encouraging Public-Private Partnership

Although government schemes and hospitals are the obvious ways to attend to most population health needs, especially in LICs/LMICs, this cannot be sustained indefinitely. To cater to the less economically privileged strata in society, a model where the private health care setups are given incentives and offered waivers and subsidies on regulations, land costs, machine procurement, and tax advantages may be necessary to promote the establishment of more RT centers. A study from Nigeria reported fewer breakdowns in RT setups with public-private partnerships than publicly funded facilities, thus enabling more patients to be treated per treatment unit. However, the public-private partnership setups had a much higher (338%) baseline capital investment.^27^ In India, the National Health Authority was formed to implement India's flagship public health insurance/assurance scheme called Ayushman Bharat Pradhan Mantri Jan Arogya Yojana. One of the primary responsibilities of the National Health Authority is the recruitment of resources (including EBRT and brachytherapy machine) from the government and private sector at competitive market rates. Additionally, it oversees engagement with all stakeholders including private sector and civil society organizations, and develops strategic partnerships in an attempt to achieve the objective of affordable health care for all.^28^

### Relaxation of Custom Duty and Other Governmental Actions

Although indigenous solutions should be encouraged, the manufacturing of LA is a complex and challenging issue, and mature, sophisticated, reliable, high-quality local replacement technology is not a quick or straightforward solution. The existing import duty on a linear accelerator in India is approximately 37% and ranges from around 20% to 40% across the globe. The practical value of taxing life-saving devices is considered retrogressive and ill-advised in most policy circles, and health administrators need to be educated and convinced about exploring options to obviate such actions. For example, if the equipment is donated, taxation would appear to mock the intent and process. If the equipment is destined for a country or region with minimal to no access, taxation should potentially be waived. A fully transparent and continually evolving certificate of need process and taxation for saturated zones could be aggressively considered to avoid oversaturation in select geographic areas and promote investment in underserved areas.

### Global Collaboration for Resource Exchange

On the basis of some estimates, the number of trained radiation oncologists may likely exceed the required number in some countries in the near future. Training programs in these countries are already facing an applicant shortfall and some might even need to close in the future. Global collaboration on this front could perhaps permit the training of personnel from underserved countries in such programs. Redistribution of workforce from countries with adequate workforce to countries with a paucity of trained workforce can be used to address the immediate crisis in certain countries in which establishment of quality training programs is likely to take time. Some HICs have even proposed the medical equivalent of the USAID Peace Corps programs to address some of these.^29,30^

### Cross-Country Collaborations

The African continent exemplifies the scenario where cross-country collaboration can provide significant breakthroughs for providing access to radiotherapy to patients with cancer. Many countries still rely on foreign aid for their health care infrastructure.^31^ Initiatives like the African Organisation for Research & Training in Cancer (AORTIC) can help smooth collaborative efforts for establishing new centers and redistributing the existing workforce within Africa.^32,33^ If the initial cost of the setup is borne by two or three agencies instead of burdening a single investor, and the number of patients requiring treatment is also pooled from a few small nations, the setup is likely to break even in terms of cost efficiency earlier than multiple centers with a limited number of patients. Transnational efforts, including forward-thinking tax and investment regulations, would catalyze such approaches.

### Training and Leveraging Online Remote Solutions

Training and research remain the mainstay of providing high-quality standardized care. Although onsite training sessions are irreplaceable, radiation oncology as a discipline benefits from distance training programs in contouring, planning, treatment delivery, and quality assurance. The recent COVID-19 pandemic has converted most in-person meetings, lectures, symposia, etc, to remote online activities with excellent efficiency and cost-savings. Lessons learned from this imposed solution could be vital in creating out-of-the-box solutions for training. A considerable component of training can be conducted remotely and through video-recorded presentations that can be delivered almost anywhere in the world with an unlimited ability for repetition and access. Such training can encompass radiation oncologists, medical physicists, dosimetrists, etc, and could significantly decrease training costs and potentially even training timelines to overcome the recognized personnel shortfall.^34,35^ It is feasible to envisage an international effort led by a global body like the IAEA or others. Telemedicine also can improve the quality of care for patients and caregivers.^36,37^

### Innovative Strategies and Research

Innovative, cost-effective research focusing on and catering to local needs should be encouraged to derive maximum benefit from existing RT facilities. For example, as the incidence of cervical carcinoma declines, a brachytherapy-sharing model between three or four proximate centers could be considered. Similarly, multiple stakeholders can acquire costly technology and operate as a common resource. On the basis of a needs assessment, a handful of centralized centers with high-end technology capabilities can be considered.

Another novel approach would be utilization of hypofractionation schedules in selected patients with cancer, categorically demonstrated to be equivalent to longer schedules; this could lead to considerable resource savings and improve access. Examples include the utilization of the short-course radiotherapy in patients with rectal cancer and moderate/extreme hypofractionation in patients with prostate and breast cancer.^38,39^ Furthermore, as extremely hypofractionated schedules such as one-five fractions come into vogue, patients can potentially travel to distant centers for relatively short durations to access life-saving therapies.

In conclusion, although the availability of RT facilities is currently suboptimal at best, this situation is likely to worsen substantially over the next two decades unless an effective and sustainable solution is achieved. A combination of global collaborative programs, country-level policymaking, and state-level implementation is required to circumvent the crisis, and all stakeholders need to be engaged. Beyond access to technology, the manpower situation in radiation oncology also needs to be addressed simultaneously in all countries, emphasizing an adequate, trained workforce. Additionally, several novel solutions, such as a global curriculum with remote access training, could be implemented to achieve this goal.
